# Parasite's Double Play Helps Evade Mouse Immune Response

**DOI:** 10.1371/journal.pbio.1001359

**Published:** 2012-07-10

**Authors:** Robin Mejia

**Affiliations:** Freelance Science Writer, Albany, California, United States of America

**Figure:**
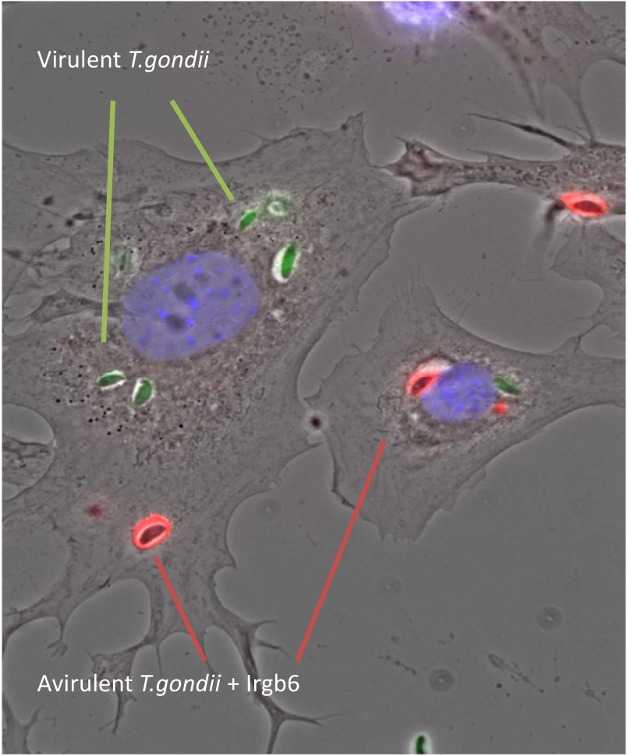
Interferon-induced mouse cells were infected simultaneously with a mixture of avirulent (black) and virulent (GFP-green) *T. gondii.* **The mouse IRG resistance protein, Irgb6 (red), is present only on avirulent strain vacuoles. Two pseudokinase proteins from the virulent strain prevent IRG proteins from loading onto the vacuole, thus saving vacuoles from destruction.** (Preparation and photo: Aliaksandr Khaminets.)

Researchers estimate that about one-third of the world's population has been infected by *Toxplasma gondii*. Most have silent infections; their immune system keeps the parasite in check and there are no clinical signs of disease. However, when *T. gondii* does cause illness, the resulting disease, toxoplamosis, can be unpleasant. Mild cases may feel like the flu, but more serious cases can result in eye infections and damage. Most importantly, pregnant women who become infected may miscarry, or the child may suffer neurological symptoms.

Except for congenital transmission, the parasite does not appear to spread between people. Human infections generally result from handling or eating raw meat or from eating food or drinking water contaminated with the oocyst form of the parasite. Cats play a key role in *T. gondii* evolution. The parasite reproduces sexually when an infected animal is eaten by a cat, which then sheds oocysts in its feces; this is why pregnant women are often advised to have someone else change their cat's litter box. Rodents, which cats eat, are therefore important intermediate hosts.

New research by Martin Fleckenstein and colleagues examines the virulence of various *T. gondii* strains, and highlights the role mice play in the evolution of the parasite. The researchers demonstrate how some *T. gondii* strains deflect the mouse immune response and result in a symptomatic infection. They elucidate for the first time the role of a pseudokinase called ROP5, which is essential for virulence.

Mice exposed to two of the three main strains of *T. gondii* develop a chronic infection with relatively mild symptoms. This is due to a class of proteins called interferon-gamma-inducible immunity-related GTPases (IRG proteins), which are key to the mouse immune response. Shortly after infection, IRGs attach to the vacuole surrounding the parasite and aid in its destruction, resulting in clearance of the parasite and thus restricting the spread of the infection.

However, infection by one of the three strains (type I) can produce serious disease in mice. Researchers knew that this virulent *T. gondii* strain secretes a protein kinase called ROP18 that inactivates the mouse IRG proteins by phosphorylating specific sites. *T. gondii* also produces a pseudokinase called ROP5 when it invades mouse cells. Both these proteins are key to the progression of disease, but how ROP5 affected virulence was not understood.

To understand ROP5's role in virulence, the researchers began by demonstrating that ROP5 binds directly to IRG proteins in *T. gondii*–infected cells. Three ROP5 isoforms exist; all were identified in this assay. Then, researchers infected mouse cells with wild-type virulent parasites, and also parasites in which either ROP5 or ROP18 was deleted. The deletion strains did not phosphorylate IRG proteins, and as a result were not virulent. That the ROP5 deletion strain was avirulent suggested a necessary role in virulence for the protein.

To demonstrate that the ROP5 loss was actually responsible for the loss of virulence in ROP5 deletion strains, the researchers took deletion strains and restored the expression of two isoforms, ROP5A_III_ and ROP5B_III_. These restored parasites were as virulent as the wild-type strains. However, as ROP5 had previously been shown to not have an inherent catalytic activity, this suggests that it acts as a co-factor for ROP18.

Finally, researchers identified where ROP5 binds to IRG proteins, and what conformational changes that binding produces. They found that ROP5 remained relatively rigid upon binding to IRG proteins—that is, they saw little change in ROP5's structure in its bound and unbound states. The IRG proteins, however, do undergo a conformational change on binding, which could explain how ROP5 facilitates binding of ROP18 to these proteins. The scientists identified a likely binding site and inactive conformation of the IRG protein. Further work using mutagenesis showed that while the ROP5 binding site is near the IRG activation site, it is clearly distinct—and that the ROP5 binding site is also clearly distinct from ROP18 targets. That is, ROP5 binding may prepare the IRG protein for the action of ROP18, but ROP5 does not mimic ROP18 activity.

Indeed, ROP5-bound IRG proteins show enhanced phosphorylation by ROP18, though the increase was not as large as seen in cells infected by the parasite. Further research is needed to determine whether ROP5 and ROP18 interact directly or whether ROP5 simply holds the IRG protein in a form more amenable to phosphorylation by ROP18. Either way, it appears to be an interesting case of a kinase losing its own enzymatic activity and evolving into a co-factor. Additionally, the researchers note that while this paper is the first to explain how ROP5 causes virulence, the pseudokinase may also act through additional pathways that have not yet been discovered.

The pathways for resistance to *T. gondii* described in mice are unlikely to mirror resistance mechanisms in humans; however, they do provide important insight into the inner workings and adaptations of this important parasite. The researchers note that as the keeping of domestic cats has increased in recent centuries, *T. gondii* evolution has likely accelerated, thus increasing the importance of understanding the parasite's evolutionary responses.

**Fleckenstein MC, Reese ML, Könen-Waisman S, Boothroyd JC, Howard JC, et al. (2012) A** ***Toxoplasma gondii*** **Pseudokinase Inhibits Host IRG Resistance Proteins. doi:10.1371/journal.pbio.1001358**

