# Correction: Parasite's Double Play Helps Evade Mouse Immune Response

**DOI:** 10.1371/annotation/a40342be-8f9e-4650-a845-1e91ff06c27c

**Published:** 2012-08-16

**Authors:** Robin Mejia

There was an error in the legend for this figure. The complete, correct figure legend is: Interferon-induced mouse cells were infected simultaneously with a mixture of avirulent (black) and virulent (GFP-green) *T. gondii.* The mouse IRG resistance protein, Irgb6 (red), is present only on avirulent strain vacuoles. A kinase and a pseudokinase from the virulent strain together prevent IRG proteins from loading onto the vacuole, thus saving vacuoles from destruction. (Preparation and photo: Aliaksandr Khaminets.)

